# Mirror Descent and Exponentiated Gradient Algorithms Using Trace-Form Entropies

**DOI:** 10.3390/e27121243

**Published:** 2025-12-08

**Authors:** Andrzej Cichocki, Toshihisa Tanaka, Frank Nielsen, Sergio Cruces

**Affiliations:** 1 Systems Research Institute of Polish Academy of Science, Newelska 6, 01-447 Warsaw, Poland; 2 Department of Electrical Engineering, Warsaw University of Technology, Koszykowa 75, 00-662 Warsaw, Poland; 3 Department of Electronic and Information Engineering, Tokyo University of Agriculture and Technology, Koganei-shi 184-8588, Japan; 4 Riken Artificial Intelligence Project (AIP), 1 Chome-4-1, Nihonbashi 103-0027, Japan; 5 Sony Computer Science Laboratories, Tokyo 141-0022, Japan; 6 Department of Signal Processing and Communications, Universidad de Sevilla, 41092 Seville, Spain

**Keywords:** mirror descent, natural gradient, information geometry, deformed logarithms, generalized exponentiated gradient, Bregman divergences, Riemnnian optimization, (*q,κ*)-algebra

## Abstract

This paper introduces a broad class of Mirror Descent (MD) and Generalized Exponentiated Gradient (GEG) algorithms derived from trace-form entropies defined via deformed logarithms. Leveraging these generalized entropies yields MD and GEG algorithms with improved convergence behavior, robustness against vanishing and exploding gradients, and inherent adaptability to non-Euclidean geometries through mirror maps. We establish deep connections between these methods and Amari’s natural gradient, revealing a unified geometric foundation for additive, multiplicative, and natural gradient updates. Focusing on the Tsallis, Kaniadakis, Sharma–Taneja–Mittal, and Kaniadakis–Lissia–Scarfone entropy families, we show that each entropy induces a distinct Riemannian metric on the parameter space, leading to GEG algorithms that preserve the natural statistical geometry. The tunable parameters of deformed logarithms enable adaptive geometric selection, providing enhanced robustness and convergence over classical Euclidean optimization. Overall, our framework unifies key first-order MD optimization methods under a single information-geometric perspective based on generalized Bregman divergences, where the choice of entropy determines the underlying metric and dual geometric structure.

*This paper is dedicated to Professor Shun-Ichi AMARI in honor of his 90th birthday.*

## 1. Introduction

Mirror descent (MD), initially proposed by Nemirovsky and Yudin [1], has become an increasingly popular topic in optimization, artificial intelligence, and machine learning domains [2,3,4,5,6]. Its profound success stems not merely from its algorithmic efficiency, but also from deep mathematical connections to information geometry and the natural statistical structure underlying optimization problems. These connections, particularly to Amari’s Natural Gradient (NG) method, reveal that effective optimization is fundamentally about respecting the intrinsic geometry of the parameter space rather than imposing artificial Euclidean constraints [7,8,9].

The central motivation for our research emerges from a fundamental insight in information geometry: optimized learning algorithms should adapt to the Fisher information metric of the underlying statistical manifold. This principle, pioneered by Amari in the context of neural networks, establishes that the steepest descent direction on a statistical manifold is not the Euclidean gradient, but rather the natural gradient, the direction that accounts for the curvature induced by the Fisher information matrix [10,11].

### 1.1. The Information Geometry Perspective

The connection between Mirror Descent and Natural Gradient runs deeper than algorithmic similarity—it represents a fundamental mathematical equivalence that has been rigorously established [2,12,13]. Our work extends this principle by showing that trace-form entropies induce natural Fisher—like metrics that can be even more appropriate for specific problem structures. Through deformed logarithms, we can systematically explore the space of possible geometries and the possibility of discovering optimal choices of hyperparameters for given data distributions [14,15].

### 1.2. Challenge of Geometric Selection

While the power of geometric optimization is well established, a fundamental challenge remains: how to select the appropriate geometry for a given optimization problem? Classical approaches require domain expertise and manual tuning, limiting their applicability. Our approach addresses this through parameterized entropy families, where hyperparameters control the geometric structure [2].

The theoretical foundation of our approach rests on the connection between Bregman divergences and exponential families established in information geometry [8]. This connection indicates that choosing a generalized entropy is equivalent to selecting an appropriate exponential family structure for specific optimization problems. See also [16] for an extension of the logarithmic divergences extending to Bregman divergences. Our deformed logarithms allow us to systematically explore the space of possible exponential families, enabling discovery of optimal statistical models.

The Exponentiated Gradient (EG) and its extensions emerge as a specific and powerful instantiation of the Mirror Descent framework when the mirror map is constructed from generalized entropies and deformed logarithms. This connection is far from superficial—it represents a fundamental mathematical relationship that unifies additive and multiplicative gradient updates within a single theoretical framework [15,17,18,19,20,21].

It is important to note that Mirror Descent updates can be reparameterized as Gradient Descent in appropriately chosen coordinate systems [2,12]. This reveals that the computational complexity of Natural Gradient can be considerably reduced. Our deformed logarithms approach gives some insight by showing that deformed logarithms naturally induce reparameterizations that preserve geometric structure while enabling efficient computation and provide implicit regularization through the choice of entropy or deformed logarithm.

### 1.3. Research Contributions and Scope

In this work, we systematically investigate the theoretical foundations and practical implications of employing trace-form entropies and deformed logarithms in the Mirror Descent framework.

Our primary contributions include:

- Mathematical Framework: We establish a comprehensive mathematical foundation connecting generalized entropies, deformed logarithms, and Mirror Descent updates, providing explicit formulations for numerous well-established trace entropy families.
- Algorithmic Innovations: We derive novel Generalized Exponentiated Gradient (GEG) algorithms with generalized multiplicative updates that leverage the flexibility of hyperparameter-controlled deformed logarithms, enabling adaptation to problem geometry.
- The significance of this research extends beyond algorithmic development; it opens new avenues for understanding the geometric foundations of optimization and provides practical tools for addressing increasingly complex machine learning challenges. The unifying theoretical framework connects optimization theory, information geometry, statistical physics, and practical machine learning, opens up new research directions, and provides principled approaches to algorithm design that respect the natural geometric structure of optimization problems.

## 2. Preliminaries: Mirror Descent (MD) and Standard Exponentiated Gradient (EG) Updates

Notations: Vectors are denoted by boldface lowercase letters, e.g., $w\in R^{N}$, where for any vector *w*, we denote its *i*-th entry by $w_{i}$. For any vectors $w,v\in R^{N}$, we define the Hadamard product as $w⊙v={\left[w_{1}v_{1},\ldots ,w_{N}v_{N}\right]}^{T}$ and $w^{\alpha }={\left[w_{1}^{\alpha },\ldots ,w_{N}^{\alpha }\right]}^{T}$. All operations for vectors like multiplications and additions are performed componentwise. The function of a vector is also applied for any entry of the vectors, e.g., $f\left(w\right)={\left[f\left(w_{1}\right),f\left(w_{2}\right),\ldots ,f\left(w_{N}\right)\right]}^{T}$. The *N*-dimensional real vector space with nonnegative real numbers is denoted by $R_{+}^{N}$. We let w(t) denote the weight or parameter vector as a function of time *t*. The learning process advances in iterative steps, where during step *t* we start with the weight vector $w\left(t\right)=w_{t}$ and update it to a new vector $w\left(t+1\right)=w_{t+1}$. We define ${\left[x\right]}_{+}=\max\left\{0,x\right\}$, and the gradient of a differentiable cost function as $\nabla_{w}L\left(w\right)=\partial L\left(w\right)/\partial w={\left[\partial L\left(w\right)/\partial w_{1},\ldots ,\partial L\left(w\right)/\partial w_{N}\right]}^{T}$. In contrast to deformed logarithms defined later, the classical natural logarithm will be denoted by ln(x).

### 2.1. Problem Statement

We consider the constrained optimization problem:

(1) $$\begin{array}{c} w_{t+1}=\underset{w\in R_{+}^{N}}{\arg\min}\left\{L\left(w\right)+\frac{1}{\eta }D_{F}\left(w|\right|w_{t})\right\}, \end{array}$$

where L(w) is a continuously differentiable loss function, η>0 is the learning rate and $D_{F}\left(w|\right|w_{t})$ is the Bregman divergence induced by a strictly convex generating function F(w) (used here as a regularizer) [2,22]. The Bregman divergence provides the geometric foundation for Mirror Descent algorithms:

(2) $$\begin{array}{c} D_{F}\left(w|\right|w_{t})=F\left(w\right)-F\left(w_{t}\right)-{\left(w-w_{t}\right)}^{T}f\left(w_{t}\right), \end{array}$$

where the generating (or potential) function F(w) is a continuously-differentiable, strictly convex function defined on the convex domain, while $f\left(w\right)=\nabla_{w}F\left(w\right)$ is the mirror map, called also the link function, which is a strictly monotonically increasing function. For fundamental properties and for some extensions, see, e.g., [23,24,25,26].

The Bregman divergence measures the difference between F(w) and its first-order Taylor approximation around $w_{t}$, providing a natural measure of geometric proximity that respects the curvature induced by *F*. This geometric structure is intimately connected to information geometry—different choices of *F* correspond to different Riemannian metrics on the parameter manifold. The Bregman divergence $D_{F}\left(w|\right|w_{t})$ arising from generating (potential) function F(w) can be viewed as a measure of curvature. The Bregman divergence includes many well-known divergences commonly used in practice, namely, the squared Euclidean distance, Kullback–Leibler divergence (relative entropy), Itakura-Saito distance, beta divergence and many more [10,14,27,28,29].

### 2.2. Mirror Descent Update Rules and Geometric Interpretation

Setting the gradient of the objective in Equation (1) to zero yields the implicit update:

(3) $$\begin{array}{c} f\left(w_{t+1}\right)=f\left(w_{t}\right)-\eta \nabla_{w}L\left(w_{t+1}\right), \end{array}$$

or equivalently

(4) $$\begin{array}{c} w_{t+1}=f^{\left(-1\right)}\left[f\left(w_{t}\right)-\eta \nabla_{w}L\left(w_{t+1}\right)\right], \end{array}$$

where $f^{\left(-1\right)}$ is inverse function of the link function. Note that when *F* is separable and continuous, the inverse function $F^{-1}$ is defined globally (by the inverse function theorem). In general, the implicit function theorem only guarantees local inversion of multivariate functions but not the existence of global inverse functions. However, when *F* is a multivariate convex function of Legendre-type so is its convex conjugate $F^{*}$ and their gradients are reciprocal to each others globally: $\nabla F={\left(\nabla F^{*}\right)}^{-1}$ and $\nabla F^{*}={\left(\nabla F\right)}^{-1}$.

Assuming that $\nabla_{w}L\left(w_{t+1}\right)\approx \nabla_{w}L\left(w_{t}\right)$, we obtain the explicit Mirror Descent update [2,5]:

(5) $$\begin{array}{c} w_{t+1}=f^{\left(-1\right)}\left[f(w_{t})-\eta \nabla_{w}L\left(w_{t}\right)\right] \end{array}$$

(6) $$\begin{array}{c} =\nabla F^{\left(-1\right)}\left(\nabla F\left(w_{t}\right)-\eta \nabla_{w}L\left(w_{t}\right)\right). \end{array}$$

In MD, we map our primal point *w* to the dual space (through the mapping via the link function f(w)=∇F(w)) and take a step in the direction given by the gradient of the function, then we map back to the primal space by using the inverse function of the link function. The advantage of using Mirror Descent (MD) over Gradient Descent is that it takes into account the geometry of the problem through suitable choice of a link function.

Dual Space Interpretation: Mirror Descent operates by

1. Mapping to dual space: Θ=f(w)=∇F(w),
2. Taking gradient step: $\Theta_{t+1}=\Theta_{t}-\eta \nabla L\left(w_{t}\right)$,
3. Mapping back to primal: $w_{t+1}=f^{-1}\left(\Theta_{t+1}\right)$.

**Figure 1:**
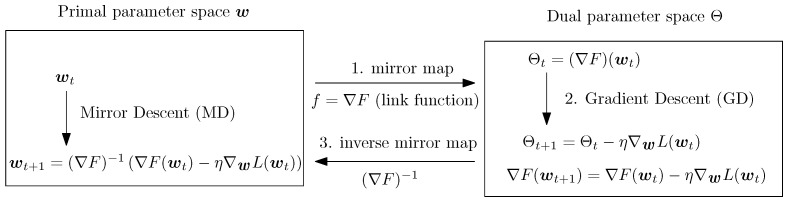
Mirror descent is a three-step process: 1. Map parameter with link function to dual space (mirror space), 2. Perform gradient descent in dual space, and 3. Maps back to primal parameter space using the inverse of the link function.

This three-step process naturally incorporates problem geometry through the choice of link function *f* (Figure 1).

For example, consider $F\left(w\right)=\sum_{i}w_{i}\log w_{i}$, the Shannon negative entropy. The link function is $f\left(w\right)=\nabla F\left(w\right)={\left[1+\log w_{i}\right]}_{i}$ with inverse map $f^{-1}\left(\Theta \right)={\left[\frac{e^{\Theta_{i}}}{\sum_{j}e^{\Theta_{j}}}\right]}_{i}$. The corresponding mirror update is the exponentiated gradient update:

$$w_{t+1}=w_{t}⊙\exp\left(-\eta \;\nabla_{w}\;L\left(w_{t}\right)\right).$$

This is a standard and useful algorithm for optimization on the probability simplex that is recovered as the mirror descent with respect to the Kullback–Leibler (KL) divergence (a Bregman divergence). The underlying geometric structure is the KL Hessian geometry, an example of dually flat space in information geometry.

Note that when the generating function *F* is separable across its coordinates (i.e., $F\left(w\right)=\sum_{i}F\left(w_{i}\right)$), the Hessian matrix $\nabla^{2}F\left(w\right)$ is diagonal.

### 2.3. Continuous-Time Formulation and Natural Gradient Connection

The continuous-time limit (as Δt→0) yields the mirror flow ODE:

(7) d f(w(t))dt=−μ∇wL(w(t)),

where μ=η/Δt>0 is the learning rate for continuous-time learning, and f(w)=∇F(w) is a suitably chosen link function [2]. Using the chain rule, we can write mirror flow as follows

(8) $$\begin{array}{c} \frac{d\;f\;\left(w\right)}{dt}=\frac{d\;f(w)}{dw}⊙\frac{d\;w}{dt}=\mathrm{diag}\left\{\frac{d\;f(w)}{dw}\right\}\frac{d\;w}{dt}=-\mu \nabla_{w}L\left(w\left(t\right)\right). \end{array}$$

Hence, we can obtain continuous-time MD update in alternative form:

(9) $$\begin{array}{c} \frac{d\;w}{dt}=-\mu \mathrm{diag}\left\{{\left(\frac{d\;f(w)}{dw}\right)}^{-1}\right\}\nabla_{w}L\left(w_{t}\right)=-\mu \;{\left[\nabla^{2}F\left(w\right)\right]}^{-1}\;\nabla_{w}L\left(w\left(t\right)\right). \end{array}$$

This reveals that Mirror Descent in continuous-time is equivalent to Natural Gradient descent with the Riemannian metric $H_{F}\left(w\right)={\left[\nabla^{2}F\left(w\right)\right]}^{-1}$. This connection, established rigorously in [2,13], shows that geometric optimization methods are fundamentally unified.

### 2.4. Discrete Natural Gradient Form

The discrete version becomes

(10) wt+1=[wt−ηdiagd f(wt)dwt−1∇wL(wt)]+

where $\mathrm{diag}\left\{{\left(\frac{d\;f(w)}{dw}\right)}^{-1}\right\}=\mathrm{diag}\left\{{\left(\frac{d\;f(w)}{dw_{1}}\right)}^{-1},\ldots ,{\left(\frac{d\;f(w)}{dw_{N}}\right)}^{-1}\right\}$, which we term Mirror-less Mirror Descent (MMD), representing a first-order approximation to second-order Natural Gradient methods (10) [30].

It should be noted that the above-defined diagonal matrix can be considered as the inverse of the Hessian matrix, if it exists and has positive diagonal entries for a specific set of parameters. The MMD is a special form of Natural Gradient Descent (NGD) [2,7,13].

F. Nielsen provided a geometric interpretation of NG and its connections with the Riemannian gradient, the mirror descent, and the ordinary additive gradient descent [31].

### 2.5. Canonical Examples and Some Geometric Insights

Case 1: For $F\left(w\right)={∥w∥}_{2}^{2}/2=\frac{1}{2}\sum_{i=1}^{N}w_{i}^{2}$ and link function $f\left(w\right)=\nabla_{w}F\left(w\right)=w$, we obtain the standard (additive) gradient descent

(11) $$\begin{array}{c} \frac{dw(t)}{dt}=-\mu_{t}\nabla_{w}L\left(w\left(t\right)\right) \end{array}$$

and its time-discrete approximate version

(12) $$\begin{array}{c} w_{t+1}=w_{t}-\eta_{t}\nabla_{w}L\left(w_{t}\right). \end{array}$$

Case 2: For $F\left(w\right)=\sum_{i=1}^{N}w_{i}\ln\left(w_{i}\right)-w_{i}$ and corresponding (componentwise) link function f(w)=ln(w) we obtain a (multiplicative) Exponentiated Gradient (EG) update also called the unnormalized EG update (or EGU) [20]:

(13) $$\begin{array}{c} \frac{d\ln\;\left(w(t)\right)}{dt}=-\mu \nabla_{w}L\left(w\left(t\right)\right),\;w\left(t\right)>0\;\;\forall \;t. \end{array}$$

In this sense, the unnormalized exponentiated gradient update (EGU) corresponds to the discrete-time version of the continuous ODE, obtained via Euler’s rule:

$$\begin{array}{cc} w_{t+1} & =\exp\left(\ln\left(w_{t}\right)-\mu \Delta t\;\nabla_{w}L\left(w_{t}\right)\right)\\ & =w_{t}⊙\exp\left(-\eta \nabla_{w}L\left(w_{t}\right)\right), \end{array}$$

where ⊙ and exp are componentwise multiplication and componentwise exponentiation, respectively, and η=μΔt>0 is the learning for discrete-time updates. This multiplicative update naturally preserves positivity constraints and corresponds to the natural geometry of the probability simplex.

### 2.6. Motivation for Using Parameterized Deformed Logarithms

Traditional Mirror Descent methods suffer from geometric rigidity—the fixed choice of mirror map *f* cannot adapt to diverse problem structures or data distributions. This limitation motivates our investigation of parameterized mirror maps based on trace-form entropies.

Adaptive Geometric Framework: Our approach addresses this fundamental limitation by introducing hyperparameter-controlled mirror maps $f_{\Theta }\left(w\right)$ that can:

- Adapt to statistical properties of training distributions.
- Interpolate between different geometries (e.g., Euclidean, exponential family, power-law).
- Provide automatic regularization through geometric bias.
- Enable systematic geometry exploration rather than ad-hoc selection.

Information-Theoretic Foundation: The connection between exponential families and Bregman divergences suggest that optimal mirror maps should reflect the underlying statistical structure of optimization problems. In fact, trace-form entropies provide systematic frameworks for discovering these optimal geometric structures.

There are many potential choices of mirror map f(w) that can model the geometry of various optimization problems and adapt to the distribution of training data. In high dimensions (large-scale optimization), it can be advantageous to abandon the Euclidean geometry to improve convergence rates and performance. Using mirror descent with an appropriately chosen function we can obtain a considerable improvement.

## 3. Why Trace Entropies and Deformed Logarithms in MD and GEG?

Entropy measures provide natural regularization mechanisms and geometric structures for optimization algorithms. The connection between entropies, information theory, and geometry runs deep; each entropy functional induces a deformed logarithm and a unique Riemannian manifold structure through its associated Fisher information metric [32,33,34,35].

Trace entropies are functionals expressible in the explicit summation form [36,37,38,39,40,41]

(14) $$\begin{array}{c} S\left(p\right)=\sum_{i}p_{i}f\left(1/p_{i}\right), \end{array}$$

where $p_{i}$ are probability values and f(·) is a suitable monotonically increasing function. The term “trace” refers to their mathematical structure, which resembles the trace operation for matrices, i.e., a direct summation over individual components.

Trace entropies are intimately connected to deformed logarithms through $f\left(x\right)=\log_{D}\left(x\right)$, where $\log_{D}$ represents a deformed logarithm function with specific mathematical properties ensuring proper entropic behavior [34,38].

A function $\log_{D}\left(x\right)$ qualifies as a deformed logarithm if it satisfies the following conditions:

- Domain $\log_{D}\left(x\right)$: $R^{+}\to R$
- Strictly monotonically increasing: $\frac{d\log_{D}\left(x\right)}{dx}>0$
- Concavity (optional): $\frac{d^{2}\log_{D}\left(x\right)}{dx^{2}}<0$
- Scaling and normalization: $\log_{D}\left(1\right)=0$, $\frac{d\log_{D}\left(x\right)}{dx}{|}_{x=1}=1$
- Duality: $\log_{D}\left(1/x\right)=-\log_{\tilde{D}}\left(x\right)$.

These axioms ensure that deformed logarithms generate well-behaved entropy functionals while providing sufficient mathematical flexibility for geometric adaptation. The concavity requirement ensures that resulting entropies satisfy the maximum entropy principle, while duality guarantees symmetric treatment of probabilities and their reciprocals, which are essential for consistent statistical interpretation.

**Remark 1.**  *It should be noted that since the generating (potential) function F(w), (which is the integral of the link function f(w)) must be strictly convex, it is sufficient that the link function $f\left(w\right)=\log_{D}\left(w\right)$ is a strictly monotonically increasing function, i.e., its first derivative must be positive but the negativity of its second derivative is in the general not necessary.*

It should be noted that deformed logarithms and their corresponding deformed exponential functions can be flexibly tuned by one or more hyperparameters, whose optimization enables the adaptation to specific data distributions and problem geometries. By tuning/learning these hyperparameters, we adapt to the distribution of training data and/or we can adjust them to achieve desired properties of gradient descent algorithms.

It is of great importance to understand the mathematical structure of the generalized logarithms and its inverse generalized exponentials in order to obtain more insight into the proposed MD or EG update schemes. Motivated by this fact, and to make this paper more self-contained, we systematically revise fundamental properties of the deformed logarithms and their inverses, generalized exponentials and investigate links between them.

We provide in the Appendix A the basics of *q*-algebra and κ-algebra and calculus in Appendix A.1 [42].

## 4. **MD and GEG Updates Using the Tsallis Entropy and Its Extensions**

### 4.1. Properties of the Tsallis *q*-Logarithm and *q*-Exponential

In physics, the Tsallis entropy is a generalization of the standard Boltzmann–Gibbs entropy [34,43,44]. It is proportional to the expectation of the deformed *q*-logarithm (referred here as the Tsallis logarithm or termed logarithm) of a distribution.

The Tsallis *q*-logarithm is defined for x>0 as [45]

(15) $$\begin{array}{c} \log_{q}^{T}\left(x\right)=\left\{\begin{array}{cc} \frac{x^{\;1-q}-1}{1-q} & \mathrm{for}\;\;x>0\;\;\mathrm{if}\;\;q\neq 1,\\ \ln(x) & \mathrm{for}\;\;x>0\;\;\mathrm{if}\;\;q=1\\ -\infty & \mathrm{for}\;\;x=0\;\;\forall q. \end{array}\right. \end{array}$$

The inverse function of the Tsallis *q*-logarithm is the deformed *q*-exponential function $\exp_{q}^{T}\left(x\right)$, defined as follows [45]:

(16) $$\begin{array}{c} \exp_{q}^{T}\left(x\right)=\left\{\begin{array}{cc} {\left[1+\left(1-q\right)x\right]}_{+}^{1/(1-q)} & \mathrm{for}\left\{\begin{array}{c} x\in (-\infty ,1/(1-q))\;\;\mathrm{if}\;\;q<1,\\ x\in (1/(q-1),\infty )\;\;\mathrm{if}\;\;q>1, \end{array}\right.\\ \exp(x) & \mathrm{for}\;\;q=1. \end{array}\right. \end{array}$$

It is easy to check that these functions satisfy the following relationships:

(17) $$\begin{array}{ccc} \log_{q}^{T}\left(\exp_{q}^{T}\left(x\right)\right) & = & x,\;\;(0<\exp_{q}^{T}\left(x\right)<\infty ), \end{array}$$

(18) $$\begin{array}{ccc} \exp_{q}^{T}\left(\log_{q}^{T}\left(x\right)\right) & = & x,\;\;\mathrm{for}\;\;x>0. \end{array}$$

**Remark 2.**  *The q-deformed exponential and logarithmic functions were introduced in Tsallis statistical physics in 1994 [44]. However, the q-deformation is related to the Box–Cox transformation (for q=1−λ), which was proposed in 1964 [46].*

**Figure 2:**
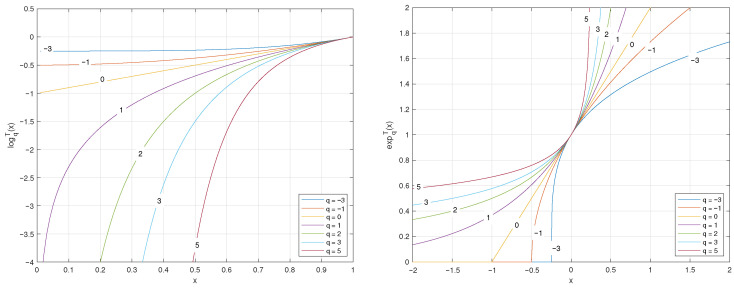
Plots of the *q*-logarithm $\log_{q}^{T}\left(x\right)$ and *q*-exponential $\exp_{q}^{T}\left(x\right)$ functions for different values of parameter *q*. From the figure, one can observe how the *q* parameter controls the degree of concavity/convexity of the *q*-logarithm as well as the degree of convexity/concavity of the *q*-exponential. Since the *q*-logarithm is convex for q<0, linear for q=0, and strictly concave for q>0, particularizing to the classical logarithm for q=1.

**Figure 3:**
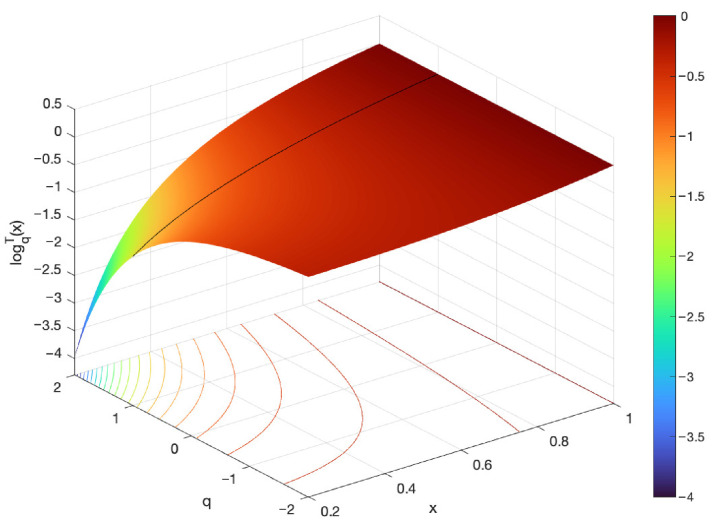
A 3D plot of the *q*-logarithm $\log_{q}^{T}\left(x\right)$. The black continuous line represents the reference of the classical logarithm ln(x), which is obtained for q=1.

The plots of the *q*-logarithm and *q*-exponential functions for various values of *q* are illustrated in Figure 2 and Figure 3.

It should be noted that *q*-functions can be approximated by power series as follows:

(19) $$\begin{array}{c} \log_{q}^{T}\left(x\right)\approx \ln\left(x\right)+\frac{1}{2}(|1-{q|)\left(\ln\left(x\right)\right)}^{2}+\frac{1}{6}{\left(1-q\right)}^{2}{\left(\ln\left(x\right)\right)}^{3}+\cdots , \end{array}$$

and

(20) $$\begin{array}{ccc} \exp_{q}^{T}\left(x\right) & \approx & 1+x+\frac{1}{2}qx^{2}+\frac{1}{6}\left(2q^{2}-q\right)x^{3}\cdots \end{array}$$

(21) $$\begin{array}{cc} & =\exp\left(x\right)+\frac{1}{2}\left(q-1\right)x^{2}+\frac{1}{6}\left(2q^{2}-q-1\right)x^{3}+O\left(x^{4}\right). \end{array}$$

These functions have the following basic properties [44,47,48,49]:

(22) $$\begin{array}{ccc} \log_{q}^{T}\left(x\right) & = & -\log_{2-q}^{T}\left(1/x\right) \end{array}$$

(23) $$\begin{array}{ccc} \frac{\partial \log_{q}^{T}\left(x\right)}{\partial x} & = & \frac{1}{x^{q}}>0 \end{array}$$

(24) $$\begin{array}{ccc} \frac{\partial^{2}\log_{q}^{T}\left(x\right)}{\partial x^{2}} & = & \frac{-q}{x^{q+1}}<0\;\;\mathrm{for}\;\;q>0. \end{array}$$

It is easy to prove the following fundamental properties:

(25) $$\begin{array}{c} \log_{q}^{T}\left(xy\right)=\log_{q}^{T}\left(x\right)+\log_{q}^{T}\left(y\right)+\left(1-q\right)\log_{q}^{T}\left(x\right))\log_{q}^{T}\left(y\right)\;\;\mathrm{if}\;\;x>0,y>0 \end{array}$$

(26) $$\begin{array}{c} \exp_{q}^{T}\left(x\right)\exp_{q}^{T}\left(y\right)=\exp_{q}^{T}\left(x+y+\left(1-q\right)xy\right). \end{array}$$

Using these properties we can define nonlinear generalized algebraic forms *q*-sum and the *q*-product (for more details about q-algebra, see [47,50]

(27) $$\begin{array}{cccc} x{⊕}_{q}^{T}y & = & x+y+\left(1-q\right)xy,\;\left(x{⊕}_{1}^{T}y=x+y\right), & \end{array}$$

(28) $$\begin{array}{ccc} x{⊗}_{q}y & = & {\left[x^{1-q}+y^{1-q}-1\right]}_{+}^{1/(1-q)}\;\;\mathrm{if}\;\;x>0,\;\;y>0\;\left(x{⊗}_{1}^{T}y=x\;y\right). \end{array}$$

Using this notation, and definitions of *q*-exponential function (16) and *q*-logarithm (15), we can write the following formulas

(29) $$\begin{array}{c} \exp_{q}^{T}\left(x+y\right)=\exp_{q}^{T}\left(x\right){⊗}_{q}\exp_{q}^{T}\left(y\right),\\ \;\;\;\;\mathrm{for}\;\;1+(1-q)x>0,1+(1-q)y>0,1+(1-q)(x+y)>0, \end{array}$$

(30) $$\begin{array}{c} \;\exp_{q}^{T}\left(\log_{q}^{T}\left(x\right)+y\right)=x{⊗}_{q}\exp_{q}^{T}\left(y\right),\\ \;\mathrm{for}x>0,1+\left(1-q\right)y>0,x^{1-q}+\left(1-q\right)y>0. \end{array}$$

which play a key role in this paper.

### 4.2. MD and GEG Updates Using the Tsallis *q*-Logarithm

Let us assume that the link function in Mirror Descent can take the following componentwise form

(31) $$\begin{array}{c} f_{q}\left(w\right)=\log_{q}^{T}\left(w\right),\;w={\left[w_{1},\ldots ,w_{N}\right]}^{T}\in R_{+}^{N}. \end{array}$$

In this case the generating function $F\left(w\right)=\sum_{i}w_{i}\log_{q}\left(w_{i}\right)-\log_{q-1}\left(w_{i}\right)$ and the Bregman divergence is a well-known beta divergence [28]:

(32) $$\begin{array}{ccc} D_{F_{q}}(w_{t+1}\left|\right|w_{t}) & = & \sum_{i=1}^{N}w_{i,t+1}\left(\log_{q}\left(w_{i,t+1}\right)-\log_{q}\left(w_{i,t}\right)\right)-\log_{q-1}\left(w_{i,t+1}\right)+\log_{q-1}\left(w_{i,t}\right)\\ & = & \sum_{i=1}^{N}w_{i,t+1}\frac{w_{i,t+1}^{1-q}-w_{i,t}^{1-q}}{1-q}-\frac{w_{i,t+1}^{2-q}-w_{i,t}^{2-q}}{2-q}, \end{array}$$

where β=1−q,  β≠0.

Applying Equation (6) and taking into account Formula (31), we obtain a novel generalized exponentiated gradient update referred to as *q*-GEG or *q*-MD update

(33) wt+1=expqTlogqT(wt)−η∇wL(wt)=wt⊗qexpqT−η∇wL(wt))

where ${⊗}_{q}$*q*-product defined by Equation (28) is performed componentwise.

The above *q*-MD update can be written in a scalar (componentwise) form as

(34) wi,t+1=wi,t⊗qexpqT−η∇wiL(wt))=wi,t1−q+expqT(−η∇wiL(wt)))1−q−1]+1/1−q

By applying the property (31) and substituting y→y/(1−(1−q)x), we can obtain the following identity

(35) $$\begin{array}{c} \exp_{q}^{T}\left(x+y\right)=\exp_{q}^{T}\left(x\right)\;\exp_{q}^{T}\left(\frac{y}{1+(1-q)x}\right)\;\;\mathrm{for}\;\left\{\begin{array}{c} x,y\in (-\infty ,1/(1-q))\;\;\mathrm{if}\;\;q<1,\\ x,y\in (1/(q-1),\infty )\;\;\mathrm{if}\;\;q>1, \end{array}\right. \end{array}$$

Hence, we obtain a simplified generalized *q*-GEG update

(36) wi,t+1=wi,texpqT−η∇wiL(wt)wi,t1−q,

which can be written in a compact vector form

(37) wt+1=wt⊙expqT−ηt⊙∇wL(wt)

where a vector of learning rates

$$\eta_{t}={\left[\eta_{1,t},\ldots ,\eta_{N,t}\right]}^{T},$$

has entries $\eta_{i,t}=\eta /\left((1+\left(1-q\right)\log_{q}^{T}\left(w_{i,t}\right)\right)=\eta \;w_{i,t}^{q-1}$.

**Remark 3.**  *Assuming that learning rate is time variable and it is represented by a vector, i.e., $\eta \to \eta w_{t}^{1-\alpha }$, where $\eta_{t}=\eta w_{t}^{1-\alpha -\beta }$, the proposed MD update takes particular form derived and extensively experimentally tested in our recent publication [15], however using a different approach employing alpha-beta divergences [14,27].*

### 4.3. MD and EG Using Schwämmle–Tsallis (ST) Entropy

Schwämmle and Tsallis proposed two-parameter entropy [51]

(38) $$\begin{array}{c} S_{q,q^{\prime }}^{ST}\left(p\right)=\sum_{i=1}^{w}p_{i}\log_{q,q^{\prime }}^{ST}\left(1/p_{i}\right),\;\;q\neq 1,\;\;q^{\prime }\neq 1, \end{array}$$

where the deformed logarithm referred to as the ST-logarithm or ST $\left(q,q^{\prime }\right)$-logarithm is defined as

(39) $$\begin{array}{c} \log_{q,q^{\prime }}^{ST}\left(x\right)=\log_{q^{\prime }}^{T}\left({\left[x\right]}_{q}\right)=\log_{q^{\prime }}^{T}e^{\log_{q}^{T}\left(x\right)}=\frac{1}{1-q^{\prime }}\left[\exp\left(\frac{1-q^{\prime }}{1-q}\left(x^{1-q}-1\right)\right)-1\right] \end{array}$$

for x>0 and $q\neq q^{\prime }$ (typically q>1 and $q^{\prime }<1$), where ${\left[x\right]}_{q}=\exp\left(log_{q}\left(x\right)\right)$ and its inverse function is formulated as a two-parameter deformed exponential, $\exp_{q,q^{\prime }}\left(x\right)$

(40) $$\begin{array}{c} \exp_{q,q^{\prime }}^{ST}\left(x\right)={\left[1+\frac{1-q}{1-q^{\prime }}\ln\left(1+\left(1-q^{\prime }\right)x\right)\right]}^{1/(1-q)}. \end{array}$$

Note that if either parameter *q* or $q^{\prime }$, or both, take the value of one, the above functions simplifies to Tsallis *q*-functions (logarithm and exponential), so we can write

(41) $$\begin{array}{c} \;\log_{q,1}^{ST}\left(x\right)=\log_{1,q}^{ST}\left(x\right)=\log_{q}^{T}\left(x\right),\;\log_{1,1}^{ST}\left(x\right)=\ln\left(x\right) \end{array}$$

(42) $$\begin{array}{c} \exp_{q,1}^{ST}\left(x\right)=\exp_{1,q}^{ST}\left(x\right)=\exp_{q}^{T}\left(x\right),\;\;\exp_{1,1}^{ST}\left(x\right)=\exp\left(x\right). \end{array}$$

In the special case for $q=q^{\prime }$, we obtain

(43) $$\begin{array}{ccc} \log_{q,q}^{ST}\left(x\right) & = & \frac{1}{1-q}\left[\exp(x^{1-q}-1)-1\right]\\ & = & \frac{1}{1-q}\left[\exp\left(\left(1-q\right)\log_{q}^{T}\left(x\right)\right)-1\right],\;x>0,\;\;q>0 \end{array}$$

and

(44) $$\begin{array}{c} \exp_{q,q}^{ST}\left(x\right)={\left[\ln\left(x(1-q)+1\right)+1\right]}_{+}^{1/(1-q)}. \end{array}$$

**Figure 4:**
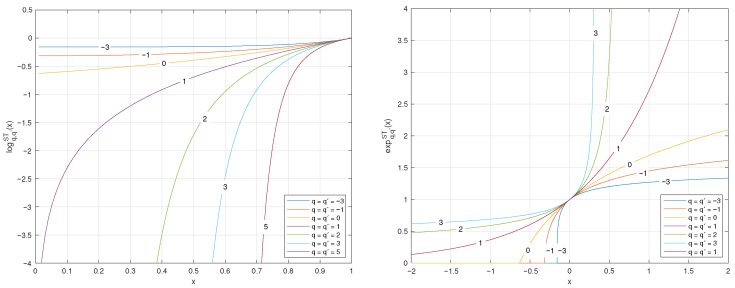
Plots of the $\left(q,q^{\prime }\right)$-logarithm and $\left(q,q^{\prime }\right)$-exponential functions for different values of the parameters in special cases when $q=q^{\prime }$.

The plots of the $\left(q,q^{\prime }\right)$-logarithm and $\left(q,q^{\prime }\right)$-exponential for various values of $q=q^{\prime }$ are illustrated in Figure 4.

Moreover, it is easy to prove the following useful properties

(45) $$\begin{array}{c} \log_{q,q^{\prime }}^{ST}\left(1/x\right)=-\log_{2-q,2-q^{\prime }}^{ST}\left(x\right), \end{array}$$

(46) $$\begin{array}{c} \frac{d\log_{q.q^{\prime }}^{ST}\left(x\right)}{dx}=x^{-q}\exp\left(\frac{1-q^{\prime }}{1-q}\left(x^{1-q}-1\right)\right)>0\;\;\mathrm{for}\;\;\;x>0,\;\;\;\forall q,q^{\prime },\;\;q\neq 1. \end{array}$$

Defining $\left(q,q^{\prime }\right)$-product as [52]:

(47) $$\begin{array}{c} x{⊗}_{q,q^{\prime }}^{ST}y=\exp_{qq^{\prime }}\left(\log_{q,q^{\prime }}\left(x\right)+\log_{q,q^{\prime }}\left(y\right)\right) \end{array}$$

we have the key formulas

(48) $$\begin{array}{ccc} \exp_{q,q^{\prime }}^{ST}\left(x+y\right) & = & \exp_{q,q^{\prime }}^{ST}\left(x\right)\;\;{⊗}_{q,q^{\prime }}\;\;\exp_{q,q^{\prime }}^{ST}\left(y\right), \end{array}$$

(49) $$\begin{array}{ccc} \exp_{q,q^{\prime }}^{ST}\left(\log_{q,q^{\prime }}^{ST}\left(x\right)+y\right) & = & x\;\;{⊗}_{q,q^{\prime }}\;\;\exp_{q,q^{\prime }}^{ST}\left(y\right). \end{array}$$

Let us consider now that the link function is defined componentwise as

(50) $$\begin{array}{c} f_{q,q^{\prime }}\left(w\right)=\log_{q,q^{\prime }}^{ST}\left(w\right). \end{array}$$

The novel $\left(q,q^{\prime }\right)$-GEG update can take the following form

(51) wt+1=wt⊗q,q′expq,q′ST−η∇wL(wt)),

In this case, the update is more complex than in the previous case. An alternative approach is to apply the MMD/NG Formula (10):

(52) wt+1=[wt−ηdiag{wq⊙exp1−q′1−q(1−w1−q)}∇wL(wt)]+

which can be written equivalently in a scalar form as

(53) wi,t+1=wi,t−ηwi,tqexp1−q′1−q(1−wi,t1−q)∂L(wt)∂wi]+

**Remark 4.** 
**Extension to Three Parameters $\left(q,q^{\prime },r\right)$-Logarithm** *. Note that by using definition ${\left[x\right]}_{q}=\exp\left(\log_{q}^{T}\left(x\right)\right)$ we can write the ST logarithm in a compact form*

(54) $$\begin{array}{c} \log_{q,q^{\prime }}^{ST}\left(x\right)=\frac{{\left[x\right]}_{q}^{1-q^{\prime }}-1}{1-q^{\prime }}=\frac{[\exp{\left(\log_{q}^{T}\left(x\right)\right]}^{1-q^{\prime }}-1}{1-q^{\prime }} \end{array}$$

*Analogously, we can define*

(55) $$\begin{array}{c} {\left[x\right]}_{q,q^{\prime }}=\exp\left(\log_{q,q^{\prime }}^{ST}\left(x\right)\right). \end{array}$$

*Hence, we can formulate a three-parameter logarithm as proposed in [53]*

(56) $$\begin{array}{c} \log_{q,q^{\prime },r}^{CC}\left(x\right)=\frac{{\left({\left[x\right]}_{q,q^{\prime }}^{1-q^{\prime }}\right)}^{1-r}-1}{1-r}=\frac{(\exp{\left(\log_{q,q^{\prime }}^{ST}\left(x\right)\right)}^{1-r}-1}{1-r} \end{array}$$

**Figure 5:**
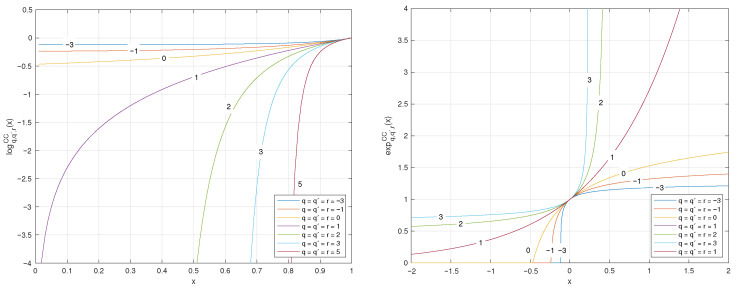
Plots of the $\left(q,q^{\prime },r\right)$-logarithm and $\left(q,q^{\prime },r\right)$-exponential functions when the parameters are coincident $q=q^{\prime }=r$.

The plots of the $\left(q,q^{\prime },r\right)$-logarithm and $\left(q,q^{\prime },r\right)$-exponential for coincident values of *q*, $q^{\prime }$, and *r* are illustrated in Figure 5.

In a similar way, as in the previous section, we can drive MD updates using as a link function the above-defined three-parameter logarithm.

## 5. MD and GEG Using the Kaniadakis Entropy and Its Extensions and Generalizations

### 5.1. Basic Properties of κ-Logarithm and κ-Exponential

An entropic structure emerging in the context of special relativity, is the one defined by Kaniadakis [36,37] as follows

(57) $$\begin{array}{c} S_{\kappa }\left(p_{i}\right)=\sum_{i}p_{i}\log_{\kappa }^{K}\left(1/p_{i}\right), \end{array}$$

where a deformed κ-logarithm referred to as the Kaniadakis κ-logarithm is defined as [36,37]:

(58) $$\begin{array}{c} \log_{\kappa }^{K}\left(x\right)=\left\{\begin{array}{cc} \frac{x^{\kappa }-x^{-\kappa }}{2\kappa }=\frac{1}{\kappa }\sinh\left(\kappa \ln\left(x\right)\right) & \mathrm{if}\;\;x>0\;\;\mathrm{and}\;\;0<\kappa^{2}<1\\ \ln(x) & \mathrm{if}\;\;x>0\;\;\mathrm{and}\;\;\kappa =0. \end{array}\right. \end{array}$$

The inverse function of the Kaniadakis κ-logarithm is the deformed exponential function $\exp_{\kappa }^{K}\left(x\right)$, represented as

(59) $$\begin{array}{ccc} \exp_{\kappa }^{K}\left(x\right) & = & \exp\left(\int_{0}^{x}\frac{dy}{\sqrt{1+\kappa^{2}y^{2}}}\right)\\ & = & \left\{\begin{array}{cc} {\left(\sqrt{1+\kappa^{2}x^{2}}+\kappa x\right)}^{1/\kappa }=\exp\left(\frac{1}{\kappa }\;\mathrm{arsinh}\left(\kappa x\right)\right) & -1<\kappa <1\\ \exp(x) & \kappa =0. \end{array}\right. \end{array}$$

**Figure 6:**
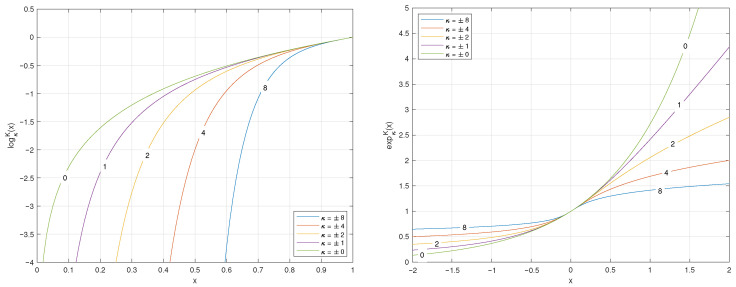
Plots of the κ-logarithm and κ-exponential functions for different values of the parameter κ.

**Figure 7:**
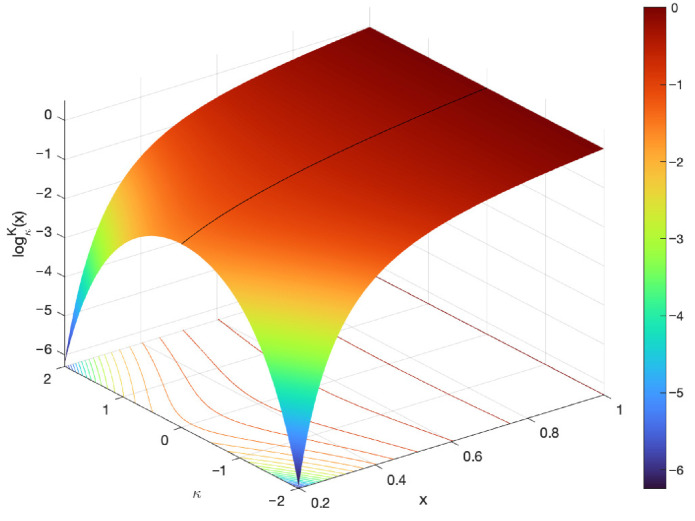
Surface plots of the κ-logarithm. The black continuous line represents the reference of the classical logarithm ln(x), which is obtained for κ=0.

The plots of the κ-logarithm and κ-exponential functions for various values of κ are illustrated in Figure 6 and Figure 7.

Note that the Kaniadakis logarithm can also be expressed in terms of the Tsallis logarithm as

(60) $$\begin{array}{c} \log_{\kappa }^{K}\left(x\right)=\frac{\log_{1+\kappa }^{T}\left(x\right)+\log_{1-\kappa }^{T}\left(x\right)}{2}. \end{array}$$

These functions have the following fundamental and useful properties [37,41]:

(61) $$\begin{array}{ccc} \;\log_{\kappa }^{K}\left(1\right) & = & 1,\;\log_{\kappa }^{K}\left(0^{+}\right)=-\infty ,\;\log_{\kappa }^{K}\left(\infty \right)=+\infty , \end{array}$$

(62) $$\begin{array}{ccc} \log_{\kappa }^{K}\left(1/x\right) & = & -\log_{\kappa }^{K}\left(x\right), \end{array}$$

(63) $$\begin{array}{ccc} \;\log_{\kappa }^{K}\left(x^{\lambda }\right) & = & \lambda \;\log_{\lambda \;\kappa }^{K}\left(x\right), \end{array}$$

(64) $$\begin{array}{ccc} \;\log_{\kappa }^{K}\left(x\;y\right) & = & y^{\kappa }\log_{\kappa }^{K}\left(x\right)+x^{-\kappa }\log_{\kappa }^{K}\left(y\right), \end{array}$$

(65) $$\begin{array}{ccc} \log_{\kappa }\left(\exp\left(x\right)\right) & = & (1/\kappa )\sinh(\kappa x), \end{array}$$

(66) $$\begin{array}{ccc} \;\ln(\exp_{\kappa }\left(x\right)) & = & (1/\kappa )\mathrm{arsinh}(\kappa x), \end{array}$$

(67) $$\begin{array}{ccc} \;\frac{\partial \log_{\kappa }^{K}\left(x\right)}{\partial x} & = & \frac{x^{\kappa }+x^{-\kappa }}{2x}=\frac{\cosh[\kappa \ln(x)]}{x}>0, \end{array}$$

(68) $$\begin{array}{ccc} \;\frac{\partial^{2}\log_{\kappa }^{K}\left(x\right)}{\partial x^{2}} & = & \frac{\kappa -1}{2}x^{\kappa -2}-\frac{\kappa +1}{2}x^{-\kappa -2}<0\;\;\mathrm{for}\;\;\kappa \in \left[-1,1\right]. \end{array}$$

The last two properties indicate that the Kaniadakis κ-logarithm is monotonically increasing for any value of κ, and for |κ|<1, it is additionally a strictly concave function.

The κ-logarithm can be approximated as a power series

(69) $$\begin{array}{c} \log_{\kappa }\left(x\right)\approx \ln\left(x\right)+\frac{1}{3!}\kappa^{2}{\left[\ln\left(x\right)\right]}^{3}+\frac{1}{5!}\kappa^{4}{\left[\ln\left(x\right)\right]}^{5}+\frac{1}{7!}\kappa^{6}{\left[\ln\left(x\right)\right]}^{7}\cdots ,\;\;x>0. \end{array}$$

Furthermore, it is important to note that applying the Taylor series expansion of the κ-exponential we can obtain a simple approximation as

(70) $$\begin{array}{ccc} \;\;\;\;\exp_{\kappa }^{K}\left(x\right) & = & 1+x+\frac{1}{2!}x^{2}+\frac{1}{3!}\left(1-\kappa^{2}\right)x^{3}+\frac{1}{4!}\left(1-4\kappa^{4}\right)x^{4}+\cdots \end{array}$$

(71) $$\begin{array}{cc} & =\exp\left(x\right)-\frac{1}{3!}\kappa^{2}x^{3}-\frac{1}{4!}4\kappa^{4}x^{4}+\cdots . \end{array}$$

Two notable features of the κ-exponential function are that it asymptotically approaches a regular exponential function for small *x* and asymptotically approaches a power law for large value |x| [37,54]. Specifically,

(72) $$\begin{array}{c} \lim_{x\to 0}\exp_{\kappa }\left(x\right)∼\;\exp\left(x\right), \end{array}$$

(73) $$\begin{array}{c} \lim_{x\to \pm \infty }\exp_{\kappa }\left(x\right)∼\;{\left|2\kappa x\right|}^{\pm 1/|\kappa |}. \end{array}$$

The κ-exponential function has the following basic properties [36,37,41]

(74) $$\begin{array}{ccc} \;\;\;\;\;\;\;\;\;\;\;\;\;\;\;\;\;\;\exp_{\kappa }^{K}\left(0\right) & = & 1,\;\;\exp_{\kappa }^{K}\left(-\infty \right)=0^{+},\;\;\exp_{\kappa }^{K}\left(\infty \right)=+\infty , \end{array}$$

(75) $$\begin{array}{ccc} \;\exp_{\kappa }^{K}\left(-\infty \right) & = & 0^{+},\;\;\exp_{\kappa }^{K}\left(+\infty \right)=+\infty , \end{array}$$

(76) $$\begin{array}{ccc} \exp_{\kappa }^{K}\left(x\right)\exp_{\kappa }^{K}\left(-x\right) & = & 1, \end{array}$$

(77) $$\begin{array}{ccc} \;{\left[\exp_{\kappa }^{K}\left(x\right)\right]}^{r} & = & \exp_{\kappa /r}^{K}\left(rx\right),\;\;r\in R, \end{array}$$

(78) $$\begin{array}{ccc} \;\frac{\partial \exp_{\kappa }^{K}\left(x\right)}{\partial x} & > & 0, \end{array}$$

(79) $$\begin{array}{ccc} \;\frac{\partial^{2}\exp_{\kappa }^{K}\left(x\right)}{\partial x^{2}} & >0 & \;\;\mathrm{for}\;\;\kappa \in [-1,1]. \end{array}$$

The last two properties mean that the Kaniadakis κ-exponential is a monotonically increasing and convex function for a specific range of the parameter κ.

The property (79) emerges as a particular case of the more general one

(80) $$\begin{array}{ccc} \exp_{\kappa }^{K}\left(x\right)\exp_{\kappa }^{K}\left(y\right) & = & \exp_{\kappa }^{K}\left(x\right){⊕}_{\kappa }\exp_{\kappa }^{K}\left(y\right), \end{array}$$

(81) $$\begin{array}{ccc} \;\log_{\kappa }^{K}\left(xy\right) & = & \log_{\kappa }^{K}\left(x\right){⊕}_{\kappa }\log_{\kappa }^{K}\left(y\right), \end{array}$$

where κ-addition is defined as

(82) $$\begin{array}{ccc} x{⊕}_{\kappa }y & = & x\;\sqrt{1+\kappa^{2}y^{2}}+y\;\sqrt{1+\kappa^{2}x^{2}} \end{array}$$

(83) $$\begin{array}{cc} & \approx x+y+\frac{\kappa^{2}}{2}\left(xy^{2}+x^{2}y\right)-\frac{\kappa^{4}}{8}\left(xy^{4}+x^{4}y\right)\cdots \end{array}$$

By defining and evaluating the κ-product

(84) $$\begin{array}{ccc} x{⊗}_{\kappa }y & = & \exp_{\kappa }\left[\log_{\kappa }\left(x\right)+\log_{\kappa }\left(y\right)\right] \end{array}$$

(85) $$\begin{array}{cc} \; & ={\left(\frac{x^{\kappa }-x^{-\kappa }}{2}+\frac{y^{\kappa }-y^{-\kappa }}{2}+\;\sqrt{1+{\left(\frac{x^{\kappa }-x^{-\kappa }}{2}+\frac{y^{\kappa }-y^{-\kappa }}{2}\right)}^{2}}\right)}^{1/\kappa } \end{array}$$

(86) $$\begin{array}{cc} & =\exp\left(\frac{1}{\kappa }\mathrm{arsinh}\left(\left(x^{\kappa }-x^{-\kappa }+y^{\kappa }-y^{-\kappa }\right)/2\right)\right) \end{array}$$

(87) $$\begin{array}{cc} & =\frac{1}{\kappa }\sinh\left(\frac{1}{\kappa }\mathrm{arsinh}\left(\kappa x\right)\;\mathrm{arsinh}\left(\kappa y\right)\right), \end{array}$$

we have the key formulas for our MD application

(88) $$\begin{array}{c} \exp_{\kappa }^{K}\left(x+y\right)=\exp_{\kappa }^{K}\left(x\right){⊗}_{\kappa }\exp_{\kappa }^{K}\left(y\right), \end{array}$$

(89) $$\begin{array}{c} \exp_{\kappa }^{K}\left(\log_{\kappa }^{K}\left(x\right)+y\right)=x{⊗}_{\kappa }\exp_{\kappa }^{K}\left(y\right). \end{array}$$

Appendix A.2 gives an overview of the κ-algebra and calculus.

### 5.2. MD and GEG Using the Kaniadakis Entropy and κ-Logarithm

Let us assume that the link function in Mirror Descent can take the following componentwise form

(90) $$\begin{array}{c} f_{\kappa }\left(w_{t}\right)=\log_{\kappa }^{K}\left(w_{t}\right),\;w={\left[w_{1,t},\ldots ,w_{N,t}\right]}^{T}\in R_{+}^{N}. \end{array}$$

Note that since the first derivative of $\log_{\kappa }^{K}\left(w\right)$ is positive and second derivative is negative, the link function is a (componentwise) increasing function and additionally a concave function for κ∈(−1,1]. In this case the generating function:

(91) $$\begin{array}{c} F_{\kappa }\left(w_{t}\right)=\sum_{i=1}^{N}\frac{1}{2\kappa }\left[\frac{w_{i,t}^{1+\kappa }}{1+\kappa }-\frac{w_{i,t}^{1-\kappa }}{1-\kappa }\right]. \end{array}$$

Taking into account Formula (6), we obtain a novel κ-GEG update

(92) wt+1=wt ⊗κ expκK−η∇wL(wt))

where ${⊗}_{\kappa }$ is a κ-product defined by Equation (87) and is performed componentwise.

### 5.3. Two-Parameter Logarithms Based on Generalized Kaniadakis–Lissia–Scarfone Entropy

Another important generalized entropy has the following form

(93) $$\begin{array}{c} S_{\kappa ,r}\left(p\right)=-\sum_{i=1}^{w}{\left(p_{i}\right)}^{r+1}\frac{p_{i}^{\kappa }-p_{i}^{-\kappa }}{2\kappa }=-\sum_{i=1}^{w}p_{i}\log_{\kappa ,r}\left(p_{i}\right), \end{array}$$

which was introduced by Sharma, Taneja and Mittal (STM) in [55,56,57], and also investigated, independently, by Kaniadakis, Lissia and Scarfone (KLS) in [38,39,58].

Equation (93) mimics the expression of the Boltzmann–Gibbs entropy by replacing the standard natural logarithm ln(x) with the two-parametric deformed logarithm $\log_{\kappa ,r}\left(x\right)$ defined as

(94) $$\begin{array}{c} \log_{\kappa ,r}\left(x\right)=x^{r}\;\frac{x^{\kappa }-x^{-\kappa }}{2\kappa },\;\;x>0,\;\;r\in R,\;\;\;\;\mathrm{for}\;\;\;-|\kappa |\leq r\leq 1/2-|1/2-|\kappa ||. \end{array}$$

**Figure 8:**
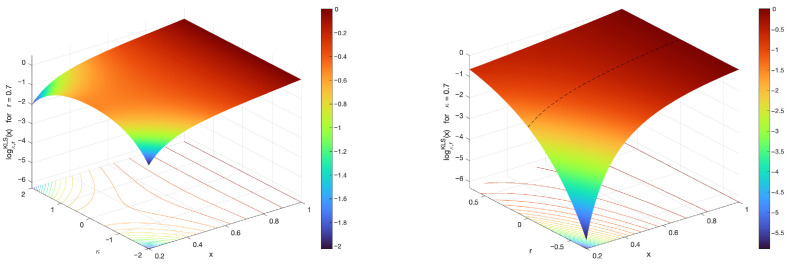
Surface plots of the (κ,r)-logarithm for various values of hyperparameters κ and *r*. The left-hand-side figure illustrates the (κ,r)-logarithm in terms of κ and *x* when r=0.7. The right-hand-side figure illustrates the (κ,r)-logarithm, now in terms of *r* and *x*, when κ=0.7. The black dashed line coincides with the κ-logarithm for κ=0.7, since in this case r=0.

**Figure 9:**
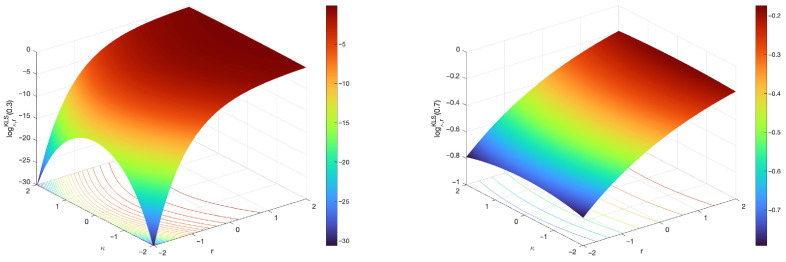
Surface plots of the (κ,r)-logarithm in terms of the hyperparameters κ and *r*, when x∈{0.3,0.7}. From the drawings, it is apparent how the changes of the hyperparameter *r* have a much stronger influence in the magnification of the response, in comparison with the changes in the hyperparameter κ that correspond with more subtle elongations. The figure on the left-hand-side evaluates the (κ,r)-logarithm for x=0.3, whereas the figure on the right-hand-side evaluates it for x=0.7.

The surface plots of the (κ,r)-logarithm for various values of hyperparameters κ and *r* are illustrated in Figure 8 and Figure 9.

The (κ,r)-logarithm can be expressed via the Kaniadakis κ-logarithm and the Tsallis *q*-logarithm

(95) $$\begin{array}{c} \log_{\kappa ,r}\left(x\right)=x^{r}\;\frac{x^{\kappa }-x^{-\kappa }}{2\kappa }=x^{r}\;\log_{\kappa }^{K}\left(x\right)=x^{r-\kappa }\log_{1-2\kappa }^{T}\left(x\right). \end{array}$$

Obviously, for r=0 the (κ,r)-logarithm simplifies to a Kaniadakis κ-logarithm, and for r=±|κ| one recovers the Tsallis *q*-logarithm with q=1∓2|κ|, (0<q<2).

By introducing a new parameter ω=r/κ or replacing r=ω κ we can represent the logarithm as

(96) $$\begin{array}{c} \log_{\kappa ,\omega }\left(x\right)=\frac{x^{\kappa (\omega +1)}-x^{\kappa (\omega -1)}}{2\kappa }, \end{array}$$

which for ω=0 simplifies to κ-logarithm and with ω=1 and κ=(1−q)/2 we have a *q*-logarithm. This formula indicates that this logarithm smoothly interpolates between a Kaniadakis logarithm and Tsallis logarithm.

Summarizing, the (κ,r)-logarithm can be described as follows [38,39]

(97) $$\begin{array}{c} \log_{\kappa ,r}^{KLS}\left(x\right)=\left\{\begin{array}{cc} \frac{x^{r+\kappa }-x^{r-\kappa }}{2\kappa } & \mathrm{if}\;\;x>0\;\;\mathrm{for}\;\;r\in R,\;\;\mathrm{and}\;\;\;-|\kappa |\leq r\leq |\kappa |,\\ \log_{\kappa }^{K}\left(x\right)=\frac{x^{\kappa }-x^{-\kappa }}{2\kappa } & \mathrm{if}\;\;x>0\;\;\mathrm{for}\;r=0,\;\kappa \in [-1,1],\;\;\kappa \neq 0,\\ \log_{q}^{T}\left(x\right)=\frac{x^{1-q}-1}{1-q}, & \mathrm{if}\;\;x>0\;\;\mathrm{for}\;r=\kappa =(1-q)/2,\;\;q\neq 1,\;q>0,\\ \ln(x) & \mathrm{if}\;\;x>0\;\;\mathrm{and}\;\;r=\kappa =0. \end{array}\right. \end{array}$$

It should also be noted the (κ,r)-logarithm can be represented approximately by the following power series for relatively small κ:

(98) $$\begin{array}{c} \log_{\kappa ,r}^{KLS}\approx \ln\left(x\right)+\frac{1}{2}\left(2r\right){\left[\ln\left(x\right)\right]}^{2}+\frac{1}{6}\left(\kappa^{2}+3r^{2}\right){\left[\ln\left(x\right)\right]}^{3}\cdots . \end{array}$$

The (κ,r) logarithm has the following basic properties

$$\begin{array}{ccc} \log_{\kappa ,r}\left(1\right) & = & 0,\;\log_{\kappa ,r}\left(0^{+}\right)=-\infty ,\;\mathrm{for}\;r<|\kappa |,\;\log_{\kappa ,r}\left(+\infty \right)=+\infty ,\;\mathrm{for}\;r>-\left|\kappa \right|, \end{array}$$

(99) $$\begin{array}{ccc} \log_{\kappa ,r}\left(x\right) & = & -\log_{\kappa ,-r}\left(1/x\right)=\log_{-\kappa ,r}\left(x\right),\; \end{array}$$

(100) $$\begin{array}{ccc} \log_{\kappa ,r}\left(x^{\lambda }\right) & = & \lambda \;\log_{\lambda \;\kappa }\left(x\right),\; \end{array}$$

(101) $$\begin{array}{ccc} \frac{\partial \log_{\kappa ,r}\left(x\right)}{\partial x} & >0 & ,\;\;\mathrm{for}\;\;-|\kappa |\leq r\leq |\kappa |,\; \end{array}$$

(102) $$\begin{array}{ccc} \frac{\partial^{2}\log_{\kappa ,r}\left(x\right)}{\partial x^{2}} & <0 & \;\;\mathrm{for}\;\;\;-|\kappa |\leq r\leq 1/2-|1/2-|\kappa ||.\; \end{array}$$

The last two properties indicate that the (κ,r)-logarithm is a strictly monotonically increasing function for −|κ|≤r≤|κ| and additionally a concave function for −|κ|≤r≤1/2−|1/2−|κ||.

**Remark 5.** 
*Relation to the Euler Logarithm: It is interesting to note that for r+k=a and r−k=b the KLS κ,r–logarithm can be represented as the Euler logarithm [17,59]:*

(103) $$\begin{array}{c} \log_{a,b}^{Eu}\left(x\right)=\frac{x^{a}-x^{b}}{a-b},\;x>0,\;\;a<0,\;\;0<b<1, \end{array}$$

*which is related to the Borges–Rodity entropy [50,60].*

*Connection to the Schwämmle–Tsallis logarithm: By applying nonlinear transformation in (103) $x\to \exp(\log_{q}\left(x\right))$ and $a=1-q^{\prime }$, b=0, we obtain the Schwämmle–Tsallis logarithm (39).*

*Connection to the Mean Value Theorem: The function has deep connections to the Mean Value Theorem applied to power functions. For the power function $g\left(t\right)=x^{t}$, the Mean Value Theorem guarantees the existence of some parameter c∈(a,b) such that $g^{\prime }\left(c\right)=\frac{g(b)-g(a)}{b-a}$, which yields:*

(104) $$\begin{array}{c} \log_{a,b}^{EU}\left(x\right)=\frac{x^{b}-x^{a}}{b-a}=c\cdot x^{c-1}\cdot \ln\left(x\right)\;\;\mathrm{for}\;\;c\in \left(a,b\right). \end{array}$$

*Logarithmic Mean Connection: The function relates to the logarithmic mean $L\left(u,v\right)=\frac{u-v}{\ln u-\ln v}$ through the substitution $u=x^{a},v=x^{b}$. These connections provide alternative computational approaches and theoretical insights.*

*Exponential Function Theory: The underlying structure connects to exponential function differentiation rules, where $\frac{d}{dt}x^{t}=x^{t}\ln\left(x\right)$, explaining the limiting behavior observed in the analysis.*

*Computational and numerical considerations: The numerical analysis reveals several important computational aspects:*

*1.*  *Numerical Stability: The KLS logarithm becomes increasingly stable as x→1, but exhibits potential numerical instability for x values far from unity.*
*2.*  *Parameter Sensitivity: Small x values create higher sensitivity to parameter changes, requiring careful numerical handling.*
*3.*  *Convergence Properties: The limiting behavior requires special computational treatment using L’Hôpital’s rule.*

### 5.4. Exponential KLS Function and Its Properties

With the existence of $\exp_{\kappa ,r}\left(x\right)$, the inverse function of $\log_{\kappa ,r}\left(x\right)$ follows from its monotonicity in R although an explicit expression, in general, cannot be given. In other words, the inverse function can not be expressed in a closed analytical form but it can be approximated and expressed, for example, in terms of the Lambert–Tsallis $W_{q}$-functions, which are the solution of equations $W_{q}\left(z\right){\left[1+\left(1-q\right)W_{q}\left(z\right)\right]}_{+}^{1/(1-q)}=z$:

(105) $$\begin{array}{c} \exp_{\kappa ,r}\left(x\right)={\left(\frac{W_{\frac{\lambda +1}{\lambda }}\left(\lambda {\tilde{x}}^{-\lambda }\right)}{\lambda }\right)}^{-1/(2\kappa ),} \end{array}$$

where λ=(2κ)/(r+κ), $\tilde{x}=2\kappa x$ and $W_{q}$ is the Lambert–Tsallis function [61].

Another much simpler approach is to use Lagrange’s inversion theorem around 1 to obtain the following rough power series approximation (which may be sufficient for most of our applications):

(106) $$\begin{array}{ccc} \exp_{\kappa ,r}\left(x\right) & \approx & 1+x+\frac{1}{2}\left(1-2r\right)x^{2}+\left(\frac{1}{6}-r+\frac{3}{2}r^{2}-\frac{1}{6}\kappa^{2}\right)x^{3}+\cdots \end{array}$$

(107) $$\begin{array}{cc} & =\exp\left(x\right)-rx^{2}+\left(\frac{3}{2}r^{2}-r-\frac{1}{6}\kappa^{2}\right)x^{3}+O\left(x^{4}\right). \end{array}$$

Hence, we can represent a (κ,r)-exponential as follows:

(108) $$\begin{array}{c} \exp_{\kappa ,r}\left(x\right)=\left\{\begin{array}{cc} \approx \exp\left(x\right)-rx^{2}+\left(\frac{3}{2}r^{2}-r-\frac{1}{6}\kappa^{2}\right)x^{3} & \mathrm{for}\;r\in R,\;\;-|\kappa |\leq r\leq |\kappa |,\;\;|\kappa |<1,\\ \exp_{\kappa }^{K}\left(x\right)={\left[\kappa x+\sqrt{1+\kappa^{2}x^{2}}\right]}^{1/\kappa } & \;\;\mathrm{for}\;r=0,\;\kappa \in [-1,1],\;\;\kappa \neq 0,\\ \exp_{q}^{T}\left(x\right)={\left[1+\left(1-q\right)x\right]}_{+}^{1/(1-q)} & \mathrm{for}\;r=\kappa =(1-q)/2,\;\;q\neq 1,\\ \exp(x) & \mathrm{for}\;\;r=\kappa =0. \end{array}\right. \end{array}$$

Furthermore, the (κ,r)-exponential function has the following fundamental properties:

$$\begin{array}{ccc} \exp_{\kappa ,r}\left(0\right) & = & 1,\;\;\exp_{\kappa ,r}\left(-\infty \right)=0^{+},\;\mathrm{for}\;r<|\kappa |,\;\exp_{\kappa ,r}{(}_{\infty })=+\infty ,\;\;\mathrm{for}\;\;r>-\left|\kappa \right|, \end{array}$$

(109) $$\begin{array}{ccc} \exp_{\kappa ,r}\left(x\right)\exp_{\kappa ,r}\left(-x\right) & = & 1,\; \end{array}$$

(110) $$\begin{array}{ccc} {\left(\exp_{\kappa ,r}\left(x\right)\right)}^{\lambda } & = & \exp_{\kappa /\lambda ,r/\lambda }\left(\lambda x\right),\; \end{array}$$

(111) $$\begin{array}{ccc} \frac{\partial \exp_{\kappa ,r}\left(x\right)}{\partial x} & > & 0,\;\;\mathrm{for}\;\;-|\kappa |\leq r\leq |\kappa |,\; \end{array}$$

(112) $$\begin{array}{ccc} \frac{\partial^{2}\exp_{\kappa ,r}\left(x\right)}{\partial x^{2}} & > & 0,\;\;\mathrm{for}\;\;\;-|\kappa |\leq r\leq 1/2-|1/2-|\kappa ||\; \end{array}$$

The last two properties mean that the (κ,r)-exponential is a monotonically increasing convex function.

Two notable features of the (κ,r)-logarithm and exponential function are that it asymptotically approaches a regular exponential function for small *x* and asymptotically approaches a power law for large absolute *x*:

(113) $$\begin{array}{ccc} \lim_{x\to 0^{+}}\log_{\kappa ,r}\left(x\right) & ∼ & \;\frac{-1}{2|\kappa |\;x^{|\kappa |+r}}, \end{array}$$

(114) $$\begin{array}{ccc} \lim_{x\to +\infty }\log_{\kappa ,r}\left(x\right) & ∼ & \;\frac{x^{|\kappa |+r}}{2\kappa },\; \end{array}$$

(115) $$\begin{array}{ccc} \lim_{x\to 0}\exp_{\kappa ,r}\left(x\right) & ∼ & \;\exp(x),\; \end{array}$$

(116) $$\begin{array}{ccc} \lim_{x\to \pm \infty }\exp_{\kappa ,r}\left(x\right) & ∼ & {\;|2\kappa x|}^{1/(r\pm |\kappa |)}. \end{array}$$

By defining

(117) $$\begin{array}{c} x{⊗}_{\kappa ,r}y=\exp_{\kappa ,r}\left[\log_{\kappa ,r}\left(x\right)+\log_{\kappa ,r}\left(y\right)\right] \end{array}$$

we have the key formulas for our MD (GEG) implementations

(118) $$\begin{array}{ccc} \exp_{\kappa ,r}\left(x+y\right) & = & \exp_{\kappa ,r}\left(x\right){⊗}_{\kappa ,r}\exp_{\kappa ,r}^{K}\left(y\right), \end{array}$$

(119) $$\begin{array}{ccc} \exp_{\kappa ,r}\left(\log_{\kappa ,r}^{K}\left(x\right)+y\right) & = & x{⊗}_{\kappa ,r}\exp_{\kappa ,r}^{K}\left(y\right). \end{array}$$

Let us assume that the link function is defined as $f\left(w\right)=\log_{\kappa ,r}\left(w\right)$ and its inverse (if approximated version is accepted) $f^{\left(-1\right)}\left(w\right)=\exp_{\kappa ,r}\left(w\right)$, then using a general MD Formula (6), and fundamental properties described above, we obtain a general MD formula employing a wide family of deformed logarithms arising from group entropies or trace-form entropies:

(120) wt+1=expκ,rlogκ,r(wt)−ηt∇L(wt)=wt ⊗κ,r expκ,r−ηt∇L(wt))

where ${⊗}_{\kappa ,r}$-multiplication is defined/determined as follows

(121) $$\begin{array}{c} x\;{⊗}_{\kappa ,r}\;y=\exp_{\kappa ,r}\left(\log_{\kappa ,r}\left(x\right)\;+\;\log_{\kappa ,r}\left(y\right)\right). \end{array}$$

Alternatively, due to some complexity of computing precisely $\exp_{\kappa ,r}\left(x\right)$, in the general case, we can use formula MMD/NG (10) to derive a quite flexible and general NG gradient update:

(122) wt+1=[wt−ηtdiag∂logκ,r(wt)∂wt−1∇L(wt))]+

where $\mathrm{diag}\left\{{\left(\frac{\partial \log_{\kappa ,r}\left(w_{t}\right)}{\partial w_{t}}\right)}^{-1}\right\}$ is a positive-definite diagonal matrix, with the diagonal entries

(123) $$\begin{array}{c} {\left(\frac{\partial \log_{\kappa ,r}\left(w_{t}\right)}{\partial w_{i,t}}\right)}^{-1}=\frac{2\kappa }{\left(r+\kappa \right)w_{i,t}^{r+\kappa -1}-\left(r-\kappa \right)w_{i,t}^{r-\kappa -1}}>0,\;-\left|\kappa \right|\leq r\leq |\kappa |. \end{array}$$

## 6. Generalization and Normalization of Mirror Descent

Summarizing, all of the GEG updates proposed in this paper can be presented in normalized form (by projecting to unit simplex) in the following general and flexible form

(124) $$\begin{array}{ccc} \;{\tilde{w}}_{t+1} & = & w_{t}{⊗}_{D}\exp_{D}\left(-\eta_{t}⊙\nabla \hat{L}\left(w_{t}\right)\right)\;(\mathrm{Generalized}\;\mathrm{multiplicative}\;\mathrm{update}), \end{array}$$

(125) $$\begin{array}{ccc} w_{t+1} & = & \frac{{\tilde{w}}_{t+1}}{\left|\right|{\tilde{w}}_{t+1}{\left|\right|}_{1}},\;(\mathrm{Projection}\;\mathrm{to}\;\mathrm{unit}\;\mathrm{simplex}) \end{array}$$

where $\exp_{D}\left(x\right)$ ($\log_{D}\left(x\right)$) is a generalized exponential (logarithm), $\hat{L}\left(w_{t}\right)=L(w_{t}/\left|\right|w_{t}{\left|\right|}_{1})$ is normalized/scaled loss function, $\eta_{t}$ is a vector of the learning rates, $\nabla \hat{L}\left(w_{t}\right)=\nabla L\left(w_{t}\right)-\left(w^{T}\nabla L\left(w_{t}\right)\right)1$ and the generalized *D*-multiplication is computed as

(126) $$\begin{array}{c} w_{t}{⊗}_{D}\exp_{D}\left(g_{t}\right)=\exp_{D}\left(\log_{D}\left(w_{t}\right)+g_{t}\right). \end{array}$$

Here, $\log_{D}\left(w_{t}\right)$ and its inverse $\exp_{D}\left(w_{t}\right)$ mean any deformed logarithm and exponential investigated in this paper (i.e., the Tsallis, Kaniadakis, ST, KLS and KS exponential/logarithm).

Alternatively, when the inverse function can not be precisely computed, we can use an MMD/NG additive natural gradient Formula (10), which is expressed in general as

(127)wt+1=[wt−ηdiagd logD(wt)d wt−1∇wL^(wt)]+,(128)wt+1=w˜t+1||w˜t+1||1,  wt∈R+N, ∀t

where $\mathrm{diag}\left\{{\left(\frac{d\;\log_{D}\left(w\right)}{dw}\right)}^{-1}\right\}=\mathrm{diag}\left\{{\left(\frac{d\;\log_{D}\left(w\right)}{dw_{1}}\right)}^{-1},\ldots ,{\left(\frac{d\;\log_{D}\left(w\right)}{dw_{N}}\right)}^{-1}\right\}$ is a diagonal positive-definite matrix.

## 7. Conclusions and Discussion

This study establishes a comprehensive framework for applying trace-form entropies and associated deformed logarithms in both Mirror Descent and equivalently Generalized Exponentiated Gradient algorithms. By systematically exploring trace-form entropies, especially Tsallis, Kaniadakis, Scarfone, and Sharma–Taneja–Mittal forms as regularization terms, we unveil new families of mirror gradient descent algorithms that can be tailored to the optimization landscape through suitably chosen hyperparameters. The adoption of these generalized entropies opens the door to obtaining advantageous properties such as improved convergence rates, robustness against vanishing/exploding gradients, and inherent flexibility for handling non-Euclidean geometries. Table 1 summarizes the main results obtained from our study and lists the generalized exponentiated gradient update induced by the deformed exponential functions corresponding to the deformed logarithms used to define the various trace-form entropies.

The theoretical developments presented not only unify additive and multiplicative gradient update rules via Bregman divergences but also pave the way for designing robust machine learning algorithms that have the ability to adapt precisely to the structure of training data distributions via hyperparameters. Future work will investigate broader classes of entropic functions, extending the framework to non-convex and stochastic optimization settings, applying the proposed approach to practical problems, and performing systematic comparisons through computer simulation experiments.

## Figures and Tables

**Table 1:** Overview of the generalized exponentiated gradient (GEG) updates.

Entropy	Deformed Exponential	MD/GEG Update
Shannon	$\exp\left(x\right)=\sum_{i=0}^{\infty }\frac{x^{i}}{i!}$	$w_{t+1}=w_{t}⊙\exp\left(-\eta \nabla L\left(w_{t}\right)\right)$ (EG)
Tsallis	$\exp_{q}^{T}\left(x\right)=\left\{\begin{array}{cc} {\left[1+\left(1-q\right)x\right]}_{+}^{1/(1-q)} & q\neq \;1\\ \exp(x) & q=1 \end{array}\right.$	$w_{t+1}=\exp_{q}^{T}\left[\log_{q}^{T}\left(w_{t}\right){⊗}_{q}\left(-\eta \nabla_{w}L\left(w_{t}\right)\right)\right]$
		$=w_{t}{⊗}_{q}\exp_{q}^{T}\left(-\eta \nabla_{w}L\left(w_{t}\right)\right)$ (*q*-GEG)
Schwämmle-Tsallis	$\exp_{q,q^{\prime }}^{\mathrm{ST}}\left(x\right)={\left[1+\frac{1-q}{1-q^{\prime }}\ln\left(1+\left(1-q^{\prime }\right)x\right)\right]}^{1/(1-q)}$	$w_{t+1}={\left[w_{t}-\eta \mathrm{diag}\left\{w^{q}⊙\exp\left(\frac{1-q^{\prime }}{1-q}(1-w^{1-q}\right)\right\}\nabla_{w}L\left(w_{t}\right)\right]}_{+}$
Kaniadakis	$\exp_{\kappa }^{K}\left(x\right)=\left\{\begin{array}{cc} \mathrm{arcsinh}(\kappa x) & -1<\kappa <1\\ \exp(x) & \kappa =0. \end{array}\right.$	$w_{t+1}=w_{t}\;{⊗}_{\kappa }\;\exp_{\kappa }^{K}\left(-\eta \nabla_{w}L\left(w_{t}\right)\right)\left(\kappa -\mathrm{GEG}\right)$
KLS	$\exp_{\kappa ,r}\left(x\right)$	$w_{t+1}={\left[w_{t}-\eta_{t}\mathrm{diag}\left\{{\left(\frac{\partial \log_{\kappa ,r}\left(w_{t}\right)}{\partial w_{t}}\right)}^{-1}\right\}\left(\nabla L(w_{t})\right)\right]}_{+}$
Generic	$\exp_{D}\left(x\right)$	$w_{t+1}={\left[w_{t}-\eta \mathrm{diag}\left\{{\left(\frac{d\;\log_{D}\left(w_{t}\right)}{d\;w_{t}}\right)}^{-1}\right\}\nabla_{w}\hat{L}\left(w_{t}\right)\right]}_{+}$
		$w_{t+1}=\frac{{\tilde{w}}_{t+1}}{\left|\right|{\tilde{w}}_{t+1}{\left|\right|}_{1}}$

## Data Availability

No new data were created or analyzed in this study due theoretical character of this study.

## Appendix A. Deformed Algebra and Calculus

### Appendix A.1. q-Algebra and Calculus

In this section we briefly summarize *q*–Algebra [42,47]:

- *q*-sum: $x{⊕}_{q}y=x+y+\left(1-q\right)xy$
- Neutral element of *q*-sum: $x{⊕}_{q}0=0{⊕}_{q}x=x$
- *q*- substraction: $x{⊖}_{q}y=\frac{x-y}{1+(1-q)y},\;\;y\neq -1/\left(1-q\right)$
- *q*-product: $x{⊗}_{q}y={\left[x^{1-q}+y^{1-q}+1\right]}_{+}^{1/(1-q)}$
- Neutral element of *q*-product: $x{⊗}_{q}1=1{⊗}_{q}x=x$
- *q*-division: $x{⊘}_{q}y=x{⊗}_{q}\left(1/y\right)={\left[x^{1-q}-y^{1-q}+1\right]}_{+}^{1/(1-q)}$
- Inverse of *q*-product $1{⊘}_{q}x={\left[2-x^{1-q}\right]}_{+}^{1/(1-q)}$.

### Appendix A.2. κ–Algebra and Calculus

In this section we briefly summarize κ–Algebra, especially κ-product properties [36,37,38,39]:

- κ-sum: $x{⊕}_{\kappa }y=x\sqrt{1+\kappa^{2}y^{2}}+y\sqrt{1+\kappa^{2}x^{2}}$,
- Neutral element of κ-sum: $x{⊕}_{\kappa }0=0{⊕}_{\kappa }x=x$,
- κ- substraction: $x{⊖}_{\kappa }y=x\sqrt{1+\kappa^{2}y^{2}}-y\sqrt{1+\kappa^{2}x^{2}}$,
- κ-product: $x{⊗}_{\kappa }y=\exp\left(\frac{1}{\kappa }\mathrm{arsinh}\left(\left(x^{\kappa }-x^{-\kappa }+y^{\kappa }-y^{-\kappa }\right)/2\right)\right)$,
- Admits the unity as a neutral element of κ-product: $x{⊗}_{\kappa }1=1{⊗}_{\kappa }x=x$.
- κ-product is commutative: $x{⊗}_{\kappa }y=y{⊗}_{\kappa }x$
- κ-product is associative: $\left(x{⊗}_{\kappa }y\right){⊗}_{\kappa }z=x{⊗}_{\kappa }\left(y{⊗}_{\kappa }z\right)$
- The inverse element of *x* is 1/x, i.e., $x{⊗}_{\kappa }\left(1/x\right)=1$
- κ-division: $x{⊘}_{\kappa }y=x{⊗}_{\kappa }\left(1/y\right)=\exp\left(\frac{1}{\kappa }\mathrm{arsinh}\left(\left(x^{\kappa }-x^{-\kappa }-y^{\kappa }+y^{-\kappa }\right)/2\right)\right)$
- Inverse of κ-product $1{⊘}_{\kappa }x=x^{-1}$.
